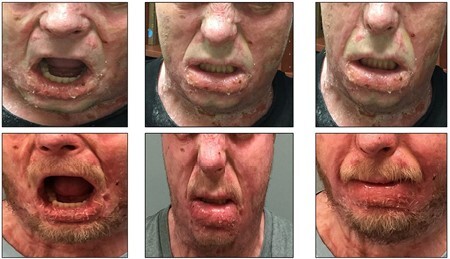# 773 Managing Facial Contractures Through Intraoral Stretching

**DOI:** 10.1093/jbcr/irae036.314

**Published:** 2024-04-17

**Authors:** Kathleen Kerr, Lori Arguello

**Affiliations:** University of Texas Medical Branch, Galveston, TX; University of Texas Medical Branch, Galveston, TX

## Abstract

**Introduction:**

Surgical management of scar tissue and contractures from facial burns has systematic approaches, yet nonsurgical management continues to lack consensus regarding therapeutic approaches capable of providing stretching of the facial skin and muscles for both treating and preventing microstomia and orofacial contractures. This exploratory study introduces an intraoral splinting system designed to provide low-load, prolonged stretching of orofacial skin and musculature to treat contractures following a facial burn injury. The treatment adjunct described here has potential application across disciplines treating microstomia and orofacial contractures.

**Methods:**

An 18-year-old male with full thickness head and neck burns participated in a 5-week trial using intraoral splints to provide stretch to areas of contracture in the mid and lower face. The trial commenced 4 ½ months after his burn injury. Prescriptive placement, sizing and time for use of the splints were provided. Changes in distance between facial landmarks were identified for 13 measurements taken for 9 facial expressions. Change between landmarks was measured in millimeters of distance between landmarks.

**Results:**

Lengthening (+measurements) or shortening (-measurements) of the distances between two landmarks indicated the potential benefit from stretching the oral cavity with intraoral splints. For the expression “open mouth wide”, increased distance between two endpoints was noted in 11 of 13 measurements, ranging from 1.6 mm to 10.8 mm. This represents a lengthening of tissue, allowing for wider oral opening. Negative measurements between two endpoints occurred in 2 of 13 measurements, demonstrating elevation of the left and right nostrils. Visual data also demonstrated the appearance of change over the 5-week trial. Pre- and post-treatment photographs are shown in Figure 1. Week 1 is shown above and week 5 is shown below. Facial expressions left to right are: open mouth wide, wrinkle nose, at rest. Figure 2 includes data from the expression "open wide".

**Conclusions:**

When treating microstomia and orofacial contractures, consideration should be given to the use and further exploration of a more targeted intraoral methodology which uses a low load and prolonged stretch to improve functional and aesthetic deficits. Oral splinting using an intraoral methodology is underutilized in the burn patient population and has application in both the acute care and chronic phases of recovery.

**Applicability of Research to Practice:**

This research methods described here are appropriate to incorporate into clinical practice patterns in both acute and post-acute rehabilitation of the patient with an orofacial burn or orofacial contractures. The methodology highlighted within this research is relevant to and has potential use for all members of the burn care team.